# Structure and function of neocortical layer 6b

**DOI:** 10.3389/fncel.2023.1257803

**Published:** 2023-09-06

**Authors:** Dirk Feldmeyer

**Affiliations:** ^1^Research Centre Jülich, Institute of Neuroscience and Medicine 10 (INM-10), Jülich, Germany; ^2^Department of Psychiatry, Psychotherapy, and Psychosomatics, RWTH Aachen University Hospital, Aachen, Germany; ^3^Jülich-Aachen Research Alliance, Translational Brain Medicine (JARA Brain), Aachen, Germany

**Keywords:** neocortex, layer 6b, synaptic connectivity patterns, neuromodulation, acetylcholine, orexin (hypocretin), neurotensin, dopamine

## Abstract

Cortical layer 6b is considered by many to be a remnant of the subplate that forms during early stages of neocortical development, but its role in the adult is not well understood. Its neuronal complement has only recently become the subject of systematic studies, and its axonal projections and synaptic input structures have remained largely unexplored despite decades of research into neocortical function. In recent years, however, layer 6b (L6b) has attracted increasing attention and its functional role is beginning to be elucidated. In this review, I will attempt to provide an overview of what is currently known about the excitatory and inhibitory neurons in this layer, their pre- and postsynaptic connectivity, and their functional implications. Similarities and differences between different cortical areas will be highlighted. Finally, layer 6b neurons are highly responsive to several neuropeptides such as orexin/hypocretin, neurotensin and cholecystokinin, in some cases exclusively. They are also strongly controlled by neurotransmitters such as acetylcholine and norepinephrine. The interaction of these neuromodulators with L6b microcircuitry and its functional consequences will also be discussed.

## Introduction

Layer 6b is thought to be a remnant of the subplate that develops during early stages of corticogenesis and persists, at least in part, into adulthood (Chen et al., 2012; Tolner et al., 2012; Marx et al., 2017). Over the past decades, many excellent reviews have been published on the subplate and its role in cortical development, neuronal migration and synaptic connectivity (for recent reviews see e.g., Kostović, 2020; Molnár et al., 2020; Chang and Kanold, 2021). In stark contrast to this, layer 6b has received much less attention and many studies on the structural and functional properties of layer 6 do not mention this sub-lamina. In a brief review, layer 6b has been described as a “cortical layer with no known function” (Molnár, 2019); however, it is briefly mentioned that this layer innervates higher order thalamic nuclei.

**FIGURE 1:**
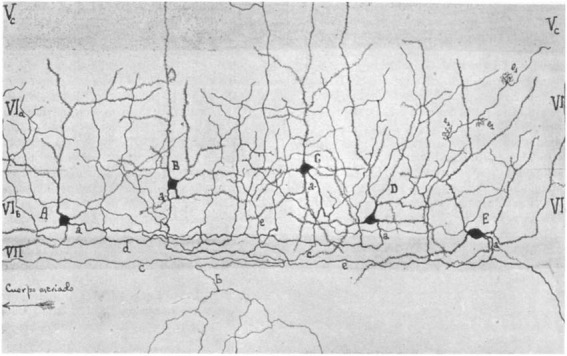
*Camera lucida* drawing of layer 6 of the barrel cortex by Lorente de Nó (1922) showing a distinct layer 6a (VI_a_) and 6b (VI_b_). Note the different types of pyramidal (A,B) and spiny non-pyramidal cells (C–E) in layer 6b. Layer VII is the white matter and is free of cell bodies in this drawing. In the original paper, the cortical area was mistaken for the auditory cortex but is indeed the first description of the somatosensory barrel cortex (Fairén et al., 1992).

Sublamina 6b of the neocortex was first described by Lorente de Nó (1922) (see also Fairén et al., 1992) as the lower part of layer 6, the layer of “polymorphous” cells, who introduced the terminology “layer 6b” (see Figure 1). It is located in the transition zone between the neocortex and the underlying white matter (WM) and is considered to be a remnant of the subplate in the adult brain (Reep and Goodwin, 1988; Clancy and Cauller, 1999; Torres-Reveron and Friedlander, 2007; Hoerder-Suabedissen and Molnár, 2015; Marx et al., 2017; Viswanathan et al., 2017). Surprisingly, this layer remains poorly characterized to date, and its functional roles after corticogenesis and in the adult brain are just beginning to emerge. In recent years, however, the functional and structural properties of layer 6b (L6b) neurons have regained interest (Hoerder-Suabedissen and Molnár, 2013; Marx and Feldmeyer, 2013; Marx et al., 2017; Hoerder-Suabedissen et al., 2018; Zolnik et al., 2020; Ben-Simon et al., 2022), and thus this review may be timely.

Layer 6b has also been variously referred to as the “subgriseal” layer (Clancy and Cauller, 1999) or layer VII/layer 7 (e.g., Marín-Padilla, 1978; Reep and Goodwin, 1988; Dediego et al., 1994; Vandevelde et al., 1996; Clancy and Cauller, 1999; Reep, 2000; Heuer et al., 2003) to emphasize its different developmental origin and its role as a structural entity separate from layer 6a (for reviews see Hoerder-Suabedissen and Molnár, 2015; Molnár et al., 2020). In recent years, the term “layer 6b” has become the accepted terminology for this layer and will be used throughout the remainder of this review; only occasionally, when referring to L6b during early postnatal development (P1-P8) the terminology subplate/layer 6b will be used.

Layer 6b has also been identified in species belonging to different mammalian species belonging to different mammalian orders. For example, in carnivores (cats and ferrets, Prieto and Winer, 1999; Rowell et al., 2010), the artiodactyl giraffe (Graïc et al., 2022), and several primate species including macaque, chimpanzee, and human (Watakabe et al., 2007; Balaram and Kaas, 2014; for a direct comparison between mouse and macaque layer 6b see Lein et al., 2017), the presence of a layer 6b has been reported but not in a number of other mammalian species. The exact relationship between layer 6b in these species and rodent layer 6b remains to be determined.

## Width of layer 6b

To date, there is no consensus on the width of sub-lamina 6b as a fraction of total layer 6, and reported values for the relative width of layer 6b vary widely, even for a given species.

Many studies, particularly in rodents, describe layer 6b as a dense band of neurons separated from the rest of the cortex by a narrow ribbon that contains only few neurons with horizontally projecting axons (e.g., Bruguier et al., 2020). In these studies the width of layer 6b is ∼50 μm (in mouse) and represents only ∼15–20% of the total layer 6 (Reep and Goodwin, 1988; Clancy and Cauller, 1999; Torres-Reveron and Friedlander, 2007).

Another set of studies defines cortical layers by the expression of specific marker genes. A number of marker genes have been identified for layer 6b in sensory cortices, e.g., the connective tissue growth factor (*Ctgf*) (Heuer et al., 2003), the orphan nuclear receptor 1 (*Nurr1*) (Liu and Baker, 1999; Arimatsu et al., 2003), neurexophilin 3 (*Nxph3*) (Beglopoulos et al., 2005), heparin sulfatase (*Sulf2*) (Nagamine et al., 2005), complexin 3 (*Cplx3*), and monooxygenase Dbh-like 1 (*MoxD1*) (Hoerder-Suabedissen et al., 2009). All these marker genes are also expressed in the subplate, indicating that L6b and the subplate are developmentally related (Luhmann et al., 2018; Bruguier et al., 2020). In rats and mice, they label a small band of neurons just above the white matter, representing ∼15% of the width of layer 6, but their expression patterns do not completely overlap. In addition, several of these genes also labeled other cortical layers, with the exception of *Ctgf*, whose expression in rodents was exclusively restricted to layer 6b. It should be noted that some of these marker genes (*Cplx3* and *Ctgf*) are expressed throughout the *entire* layer 6 of the entorhinal cortex, which belongs to the developmentally older periarchic cortex (Ben-Simon et al., 2022).

In contrast, work focusing mainly on the morphology of L6b neurons in mice and rats demonstrated that the neuronal complement in the lower 30% of layer 6 differs markedly from those in the more superficial part, i.e., layer 6a (Lorente de Nó, 1922; Valverde et al., 1989; Fairén et al., 1992, translation of Lorente de Nó, 1922; Marx and Feldmeyer, 2013; Perrenoud et al., 2013). Moreover, a recent study on L6b corticothalamic pyramidal cells in mice appear to suggest an even broader layer 6b (∼40%; Hoerder-Suabedissen et al., 2018).

The rather compact layer 6b in rat and mouse is absent in other mammalian species. In the carnivorous cat and ferret (Prieto and Winer, 1999; Rowell et al., 2010), the artiodactyl giraffe (Graïc et al., 2022), and primates such as the tree shrew, macaque, chimpanzee, and human (e.g., Balaram and Kaas, 2014), layer 6b has been found to be as broad or nearly as broad as layer 6a. As mentioned above, the relationship and genetic marker profile of layer 6b is not identical in mammals of different orders, making a direct comparison unreliable. However, comparative *in situ* hybridization studies have shown that the anatomically defined layer 6b in ferret and mouse (Rowell et al., 2010), and macaque and mouse (Watakabe et al., 2007) have overlapping, though not identical, gene expression profiles, suggesting at least some degree of similarity. In addition, in a recent transcriptomic study neurons expressing the layer 6b-specific marker *CTGF* have been identified in different primate species (Ma et al., 2022).

## Neuronal cell types and axonal projections in layer 6b

Only a few studies have focused specifically on the neuronal cell types specific to layer 6b, which–as will be shown later–is strikingly different from that of the overlying layer 6a, most of which have been carried out in the somatosensory barrel cortex and, to some extent, in other cortical areas. Systematic studies have only become available in the last decade, but it should be noted that the morphology of neurons in “layer 7” had already been described by Ramón y Cajal (horizontal cells in “layer VII” s. Fig. 375 in Ramón y Cajal, 1911) and in layer 6b of the “auditory cortex” by Lorente de Nó [s. Figure 1; Lorente de Nó, 1922; however, the “auditory cortex” was actually the somatosensory barrel cortex as identified by Fairén et al. (1992)].

### Excitatory neurons

Compared to other cortical layers, layer 6, and layer 6b in particular, is remarkable for the high morphological diversity of excitatory neuron types. This has been recognized in previous work on the structure of the neocortex. The first studies to focus explicitly on neuronal cell types in layer 6b were performed in mouse motor cortex, rat somatosensory cortex and cat auditory cortex (Meller et al., 1969; Valverde et al., 1989; Prieto and Winer, 1999). In juvenile and adult layer 6b of the motor and somatosensory cortices, several different types of spiny neurons were found, including normally oriented, inverted, horizontal and inverted pyramidal cells (PCs) as well as “polymorphous” cells, all of which had thin dendrites with spines.

**FIGURE 2:**
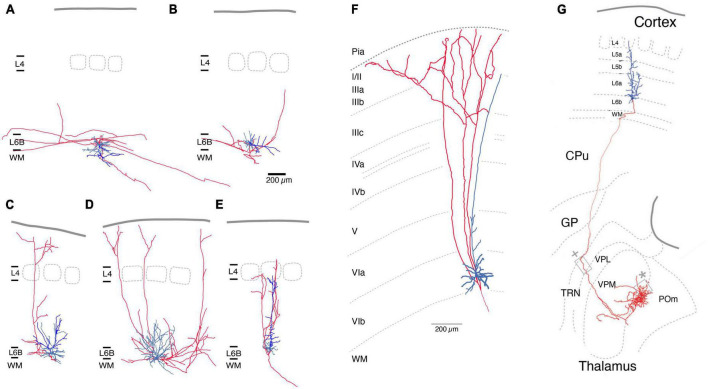
Morphological reconstructions of L6b excitatory neurons. **(A–E)** Five distinct rat L6b neuronal cell types were identified, an inverted **(A)**, a horizontal **(B)**, a tangential **(C)**, a multipolar **(D)**, and an upright pyramidal cell (PC). Of those inverted **(A)** and horizontal PCs **(B)** have horizontal axon collaterals running over long distances (>>1 mm). Rat L6b tangential PC **(C)** and multipolar excitatory neurons **(D)** have ascending axonal collaterals which arborize in layer 2/3 or 1. Normal L6b PCs with an upright apical dendrite have a narrow axonal field span in the neocortex; all axonal collaterals ascend to layers 4 up rarely beyond; the axon enters the white matter. Similarly, upright apical dendrites terminate in layer 5 [from Marx and Feldmeyer (2013); under a CC license]. **(F)** L1-projecting L6b PCs in tree shrew neocortex As in panel **(C)** the ascending axon collateral begin to bifurcate in layer 2/3 and 1. Modified from Usrey and Fitzpatrick (1996), with permission. **(G)** Mouse L6b CT PC projecting straight to the higher order Po nucleus of the thalamus without establishing collaterals in other thalamic nuclei. The L6b CT PCs have been shown to express the dopamine receptor 1 gene *Drd1*. From Hoerder-Suabedissen et al. (2018), under a CC license. Color code: Red, axon; blue, basal dendrites, bright blue main or apical dendrites (only in A-E). L1, layer 1; L6b, layer 6b; WM, white matter; CPu, caudate-putamen; CT, corticothalamic; GP, globus pallidus; TRN, thalamic reticular nucleus; VPL, ventroposterior lateral; VPM, ventral posterior medial; POm, posterior medial nucleus of the thalamus.

The axonal and dendritic domains of L6b excitatory neurons have been systematically investigated in the rat somatosensory barrel cortex (Marx and Feldmeyer, 2013). Five different excitatory neuron types were identified based on their structural and electrophysiological properties, namely PCs proper, inverted PCs, horizontal and tangential PCs, and multipolar excitatory neurons (Figures 2A–E). Part of the dendritic domain of all L6b excitatory neurons was located in the white matter, particularly the often highly branched principal or “leading” dendrite of L6b inverted PCs. The axons of L6b inverted and horizontal PCs have horizontal long-distance axonal projections across several barrel columns, but their axon remains largely within layer 6 and the white matter (Figures 2A, B). *In vivo* studies in cat auditory and mouse somatosensory cortex (Prieto and Winer, 1999; Zolnik et al., 2020) have shown that corticocortical axon collaterals can extend into neighboring cortical areas and beyond. L6b inverted PCs may be similar to the “L6 deeper” (i.e., deep layer 6a) inverted PCs of rat barrel cortex, which have a very dense and long-range axonal plexus in layer 6 (Marx and Feldmeyer, 2013). For visual cortex it has been demonstrated that all L6b excitatory neuron types except the L6b PCs proper express *Ctgf* (Allen Institute website),^1^ a highly specific marker gene for layer 6b (see above).

In contrast, axons of L6b tangential and multipolar neurons of the barrel cortex project to superficial layers (Figures 2C, D). In the visual cortex, L1-projecting L6b neurons have been identified as short or tangential PCs that give rise to axons collateralising in upper layers 2/3 and 1. These neurons may therefore be the cellular correlates of the extensive (3–4 mm) axonal projections from layer 6b to layer 1 that have been identified in sensory as well as motor and frontal cortices (Clancy and Cauller, 1999; Schuman et al., 2021). Morphological reconstructions of the axonal domain of the L6b excitatory neurons that give rise to these L1-projecting axon collaterals suggest that they are L6b tangential PCs and multipolar excitatory neurons whose axons extend >500 μm in layer 1 (Usrey and Fitzpatrick, 1996; Prieto and Winer, 1999; Marx and Feldmeyer, 2013; Peng et al., 2020). As there are no *in vivo* reconstructions of L6b neurons available and the degree of axonal truncation in brain slice preparations is likely to be high, it is difficult to assess the true L6b-L1 connectivity, which may be substantial. It should be noted that the L6b-L1 projecting neurons collateralize in two regions, layer 5/6 and 1/2, but pass through upper layer 5, 4 and lower layer 3 without branching (Figures 2A, B, F). The L6b-L1 connection is established during development (postnatal day 1–8; Ghezzi et al., 2021) and appears to persist to adulthood. It may serve as a direct, feed-forward pathway for the recruitment of L1 interneurons or the apical tufts of L5 and L2/3 PCs by L6b excitatory neurons; however, the functional role of this excitatory pathway *in vivo* is not yet understood.

In the barrel cortex, L6b PCs proper have a sparsely tufted or untufted apical dendrite that often terminates in middle to upper layer 5 but does not reach layer 4 (Figures 2E, G). Within the neocortex their axon has a small field span (<100 μm) and is predominantly vertically oriented, i.e., has one or more ascending axon collaterals. In layer 6b, PCs with upright apical dendrites oriented toward the pia project almost exclusively to the superior thalamic nucleus (for a definition of first- and higher-order thalamic nuclei s. Guillery and Sherman, 2002) i.e., in the somatosensory system, the posterior medial nucleus (POm), without sending collaterals in the first or specific ventroposterior medial nucleus (VPM) or the thalamic reticular nucleus (TRN, Figure 2F; Bourassa and Deschênes, 1995; Hoerder-Suabedissen et al., 2018). Similar observation have been made for motor and visual cortex (Hoerder-Suabedissen et al., 2018). This is in marked contrast to L6a corticothalamic (CT) PCs of which those close to the layer 5/layer 6a border innervate VPM alone, whereas those in deeper layer 6a project to both VPM *and* POm (Bourassa and Deschênes, 1995; Chevée et al., 2018; Whilden et al., 2021); these CT PCs have been shown to express the neurotensin receptor 1 gene *Ntsr1*. L6b CT PCs have been shown to express the dopamine receptor 1 gene *Drd1* (Hoerder-Suabedissen et al., 2018) and studies of the “*in vivo*” functional properties of these cells have used *Drd1*-Cre transgenic mouse lines (Ansorge et al., 2020; Breuer and Krieger, 2022) to investigate how *Drd1*+ CT PCs affect synaptic signalling in a corticothalmocortical loop.

There is evidence that *Ntsr1*-expressing PCs are also present in layer 6b although these neurons are rare (Mease et al., 2014; Grant et al., 2016; Chevée et al., 2018; Whilden et al., 2021). By analogy with *Ntsr1*-expressing L6a PCs, *Ntsr1*+ L6b PCs may innervate also first order thalamic nuclei like VPM and LGN, but this has not been conclusively shown (but see Grant et al., 2016). However, *Ntsr1* is also expressed outside layer 6 by certain WM neurons so that it may not be a reliable L6 CT PC marker (Sundberg and Granseth, 2018).

Thus L6b excitatory neuronal cell types fall into three distinct groups showing either intracortical, long-range axonal projections predominantly in layer 6 and the white matter, intracortical neurons projecting within layer 6 and to layer 1 or subcortical CT projections suggesting a high degree of intracortical connectivity (see below).

### Inhibitory interneurons

The proportion of GABAergic interneurons in the total population of L6b neurons has been found to be very low (<5%; Boon et al., 2019), even lower than that of cortical layer 4 (6.4%; Meyer et al., 2011). While the database of L6b excitatory neurons is now beginning to expand, less attention has been paid to the characterization of the structural and functional properties of L6b interneurons.

**FIGURE 3:**
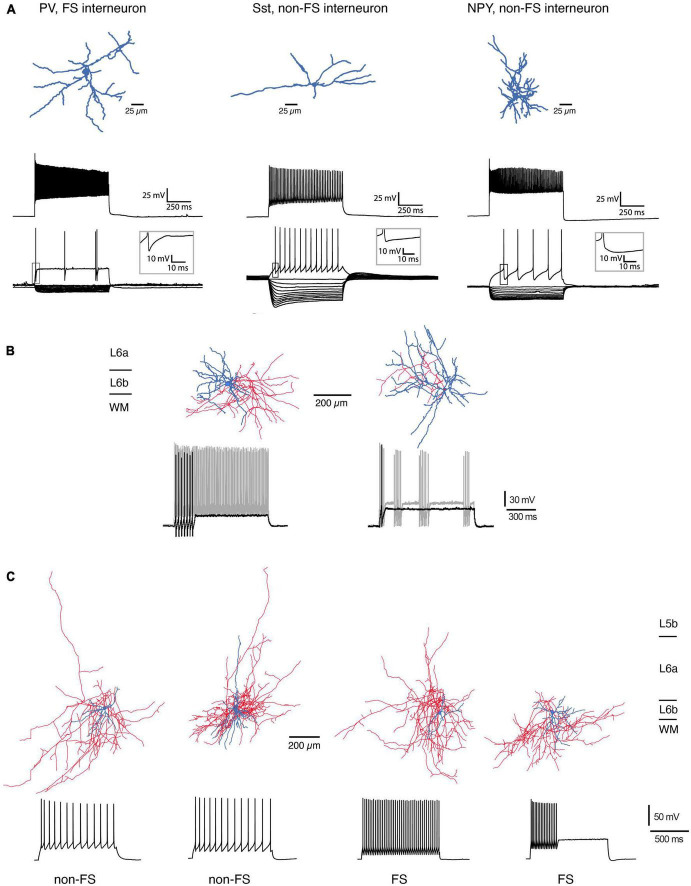
Morphological reconstructions of L6b interneurons. **(A)** Three types of L6b interneurons in mouse somatosensory barrel cortex. PV-expressing, FS interneuron, Sst+, non-FS interneuron and NPY+, non-FS interneuron. Top row, morphological reconstructions of the somatodendritic domain of the three interneuron types (blue). Middle row, Firing response during max. current injection. Bottom, voltage response during hyper- and depolarizing current steps. Note the presence of a prominent voltage sag in the Sst+ interneurons (modified after Perrenoud et al., 2013; under a CC license). **(B)** L6b FS interneurons in the rat barrel cortex may be subdivided into two types with a regular (left) and a stuttering FS firing pattern (right). The regular FS interneuron has a dense axonal domain (red) and a dendritic arbor (blue) that is largely confined to layer 6b; the stuttering FS interneuron has a broader dendritic domain with branches in layer 6a and the white matter [data from rat barrel cortex; from Marx and Feldmeyer (2013), under a CC license]. **(C)** Four types of L6b interneurons in rat prefrontal cortex (PFC). The majority of dendrites of these neurons is horizontally oriented; the axonal plexus mainly confined to layer 6. The two leftmost interneurons had the distinct adaptive spiking pattern in the non-FS cells, two rightmost are regular and stuttering FS interneurons, respectively. Note the prominent axonal plexus (blue) in the white matter most of the interneurons [from Ding et al. (2021), under a CC license].

For mouse S1 cortex, L6b neuropeptide Y (NPY)-, fast-spiking (FS) parvalbumin (PV)-, and somatostation (Sst)-expressing interneuron types have been identified based on their electrophysiological properties, dendritic morphology and marker gene expression (Perrenoud et al., 2013; Figure 3A). L6b FS interneurons in barrel cortex can be divided into two distinct groups, a regular and a stuttering type, both of which show a strong after hyperpolarisation (rFS and stFS, respectively; Marx and Feldmeyer, 2013; s. Figure 3B); of these, the latter one appears to correspond to the L6b PV+ FS interneuron described by Perrenoud and coworkers which also shows fast action potential bursts with intermittent “silent” periods.

In mouse prefrontal cortex (PFC), L6b stFS interneurons possess an elongated axonal plexus (Wenger Combremont et al., 2016a; see below) and were directly and strongly depolarized by neurotensin (NT), noradrenaline (NA), and acetylcholine (ACh) acting via nicotinic ACh receptors (nAChRs). L6b PFC FS interneurons express PV and are located close to the border between layer 6b and the white matter; they have a dense axonal plexus that projects mainly within layer 6b but sends collaterals into the white matter and layer 6a; occasionally a few collaterals also enter more superficial layers, mainly layer 5b (Ding et al., 2021). Furthermore, in rat PFC Sst+, non-FS interneurons have been identified close to the L6b/WM border (Ding et al., 2021) that have dendritic and axonal domains with prominent white matter projections; in addition these neurons send axonal projections to more superficial layers (Figure 3C). Similarly, deep layer 6 interneuron types with long-range axons entering the white matter have been found in several neocortical areas of mouse, cat, and monkey cortex [including primary and secondary somatosensory (S1,S2), secondary motor (M2), primary visual (V1), and auditory cortices (A1)]. In all these species, these neurons were immuno-positive for neuronal nitric oxide synthase (nNOS) and/or Sst and NPY (Tomioka et al., 2005; Higo et al., 2007; Tomioka and Rockland, 2007; see also Boon et al., 2019).

In mouse primary visual cortex (V1), two distinct Sst+ interneuron subtypes were found in layer 6b (Gouwens et al., 2020); however these subtypes were not restricted to layer 6b but were also identified in layer 6a. PV+ FS interneurons are also present in layer 6b of V1 [Allen Institute website (see text footnote 1)] but their morphology has not been reconstructed. Whether these neurons correspond to the L6b interneuron types described above is so far not clear.

The main feature of most L6b interneurons is their dense local dendritic and axonal arbor with few projections outside layer 6b, predominantly to layer 6a and the white matter. However, some L6b Sst+, non-FS interneurons innervate supragranular layers 2 and 3 but only sparsely. Because truncation of axon collaterals, especially long-range ones, is common in brain slice preparations, this will inevitably lead to an underestimation of the translaminar intracortical connectivity of L6b interneurons. More detailed morphological analysis in slice preparations or *in vivo* neuronal labeling will be required to address this issue.

## Connectivity

This section attempts to describe the current knowledge of pre- and postsynaptic connectivity of L6b neurons and the level of local synaptic microcircuits (<50 μm), interlaminar synaptic connections and connections between cortical areas and across brain hemispheres.

### Local

**FIGURE 4:**
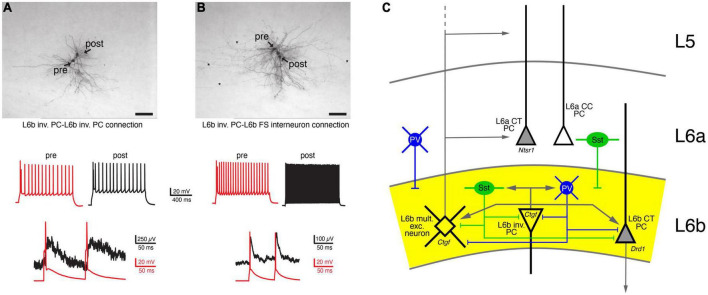
Local, intralaminar synaptic connectivity of L6b neurons. **(A)** Synaptic connection between two L6b inverted PCs (inv. PC). Top, biocytin labeling of two synaptically connected L6b inverted PCs. Middle, AP firing pattern of the presynaptic (red) and the postsynaptic neuron. Bottom, a doublet of presynaptic APs elicit weakly depressing EPSPs. **(B)** As for panel **(A)** but with a postsynaptic L6b FS interneuron. Note the fast EPSP decay in the L6b FS interneuron (Marx et al., 2017 under a CC license). **(C)** Simplified intralaminar microcircuitry of layer 6b. Synaptic inputs from layers more superficial to layer 5 and long-range connections have been omitted for clarity. L6b multipolar excitatory (L6b mult. exc.) neurons are taken as example for superficially projecting L6b neurons; inverted (inv.) PCs as examples for horizontally projecting L6b neurons. Tangential and horizontal PCs have been omitted from the diagram (but see Figure 3 and text for details). L6b CT PCs have a intracortical axonal domain so that intracortical connections are likely to be sparse; therefore, only local inputs are shown. Because of their dense local axonal domain Sst+ and PV+ interneurons are likely to contact all excitatory neurons in layer 6b. In addition, L6a Sst+ and PV+ interneurons have axonal projections into layer 6b and will therefore also innervate L6b layer excitatory neurons.

The local synaptic microcircuitry of L6b neurons, particularly within layer 6b itself and adjacent layer 6a and the white matter, is not well understood. Most of this can only be inferred from the intralaminar axonal projection pattern described above; recent rabies virus retrograde tracing study has supported this view (Zolnik et al., 2020). From this it is highly likely that L6b excitatory neurons and interneurons form local microcircuits with one another. Because of their long-range horizontal axonal projections, L6b inverted and horizontal PCs will establish synaptic connections within layer 6b, the white matter and layer 6a. It is therefore highly likely that L6b excitatory neurons and interneurons form local microcircuits with each other. Indeed, using paired recordings, it has been shown that L6b inverted PCs establish synaptic connections with other L6b neurons, but these are of relatively low efficacy (s. Figure 4; Marx et al., 2017). Similarly, it has been shown that *Drd1*- and *Ctgf*-expressing L6b excitatory neurons (putative CT and CC excitatory L6b neurons, respectively) receive little synaptic input from other *Drd1*- and *Ctgf*-expressing L6b neurons, and if they do, this input is weak (Zolnik et al., 2020).

The axonal domain of L6b CT PCs is very narrow so that these neurons will not establish a substantial number of intralaminar excitatory synaptic connections with other excitatory L6 neurons and interneurons. However, it is likely that they receive inhibitory synaptic input from both layer 6a and 6b as well as excitatory input from corticocortically projecting L6 neurons.

In contrast to excitatory neurons, both non-FS (either Sst+ or NPY+) and FS PV+ interneurons have a dense axonal plexus largely within layer 6b, but with some collaterals in layer 6a and the white matter (Marx and Feldmeyer, 2013; Gouwens et al., 2020; Ding et al., 2021). The axonal density of L6b interneurons has not been quantified yet but it is likely that the degree of intralaminar inhibitory connectivity is high. Furthermore, L6b excitatory neurons and interneurons may receive inhibitory input from different PV+ and Sst+ interneuron subtypes in layers 5 and 6a, whose axons innervate also layer 6b (Marx et al., 2017; Ding et al., 2021). To date, systematic functional studies of these connections are lacking.

L6b GABAergic interneurons (mainly of the PV+ and Sst+ subtype) are innervated by L6b excitatory neurons; an example of an L6b inverted PC-L6b FS interneuron connection is shown in Figure 4B. These connections had a low synaptic efficacy and showed weak short-term depression. Inhibitory input to layer 6b was predominantly from layer 6 (85%), particularly from layer 6b, with the remainder (15%) located in layers 4 and 5. In turn, both L6b *Drd1*+ CT PCs and *Ctgf*+ CC excitatory neurons receive inhibitory input from almost all cortical layers. Most of this input originates from PV+ and Sst+ interneurons, was relatively strong and showed short-term depression. Stimulation of VIP cells produced only small IPSPs that did not contribute to a disinhibitory mechanism as observed in L2/3 PCs. Finally, axonal afferents from layer 1 to layer 6b were identified and functional inhibitory synaptic input from L1 *Ndnf*-expressing interneuron was recorded; IPSPs at this connections were mediated by G protein-coupled GABA_*B*_ receptors (Zolnik et al., 2020).

### Interlaminar and long-range synaptic connectivity

Early studies describing dense horizontal intracortical axons emanating from layer 6b suggested that this layer gives rise to long-range corticocortical connections (Valverde et al., 1989; Prieto and Winer, 1999); however the exact targets of these axon collaterals were not identified.

### Parietal association cortex

**FIGURE 5:**
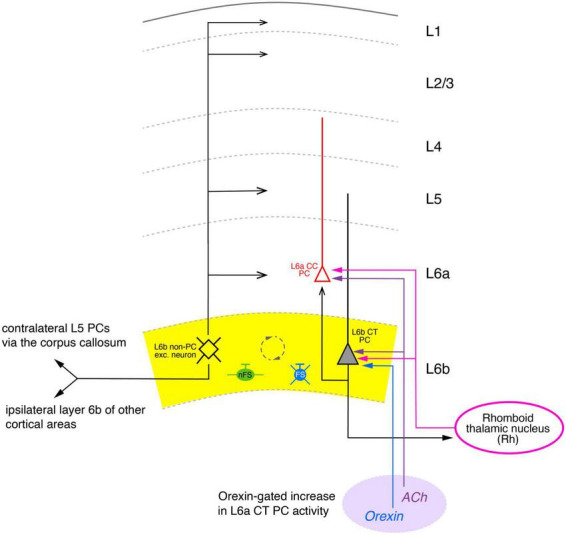
Simplified diagram of the synaptic connectivity in the parietal association cortex (PtA). The PtA L6b connectivity diagram was derived from stimulations of L6b excitatory neurons with co-applications of orexin-B and nicotine that resulted in AP firing of these neurons. In the Pta, synaptic input from multipolar PtA L6b neurons (color-coded in black) to all cortical layers was identified. Furthermore, ipsilateral long-range synaptic connections predominantly to layer 6a CC PCs (red) and 5 of adjacent cortical areas were also found. In addition, orexin/nicotine-activated synaptic input to contralateral L5 PCs was also recorded. Furthermore, L6b and L6a excitatory neurons received thalamocortical input from the Rhomboid thalamic nucleus (magenta). Furthermore, a dense network between L6b excitatory neurons, nFS (green), and L6 FS (PV+) interneurons (blue) was identified (indicated by the dashed circle). Simplified diagram of the synaptic connectivity in the parietal association (PtA) cortex. Different arrow heads signify: ▶ synaptic input to S1 layer 6b; >synaptic output from S1 layer 6b; inhibitory input is marked by bars. See main text for details).

A first attempt to reveal the medium- and long-range synaptic connectivity of L6b neurons was made by Hay and coworkers (Hay et al., 2015) in rat parietal association cortex (PtA; see Figure 5). This study was based on the finding that excitatory L6b neurons are the only neurons sensitive to orexin-B and that the orexin response is synergistically enhanced by activation of nicotinic acetylcholine receptors (nAChRs; Bayer et al., 2004; Hay et al., 2015; see below). The authors found that co-administration of nicotine and orexin resulted in an increase in local GABergic network activity.

This is indicative of a highly interconnected GABAergic network. Furthermore, an ipsilateral increase in spontaneous EPSP frequency was observed in L5 PCs, L1 interneurons and L2/3 PCs, although the effect in the latter was weak; no effect of orexin was found in L4 neurons. Thus, in the PtA L6b PCs provide strong ipsilateral inter-laminar synaptic input to layers 6, 5 and 1 but only weak and virtually no input to layers 2/3 and 4, respectively, in agreement with the morphological data (Figure 5). In addition, using a slice preparation with intact commissural axons, a profound increase in the synaptic activity in L5 PCs of the contralateral PtA was observed upon ipsilateral orexin/nicotine co-application. This is consistent with the high proportion of commissural axons in rat layer 6b reported previously (50%; Arimatsu and Ishida, 2002). Optogenetic stimulation of the higher order thalamic nucleus innervating layer 6 of the PtA, the rhomboid nucleus (Rh), allowed the characterization of the thalamocortical (TC) Rh-L6 pathway. Both L6a and L6b neurons were found to receive monosynaptic TC input from the Rh; in addition, L6a neurons received also disynaptic, feed-forward input via a Rh-L6b-L6a connection (Figure 5). It has been proposed that this results in an L6b-mediated, orexin-gated, enhancement of the reliability and temporal precision of the monosynaptic L6a-Rh input, suggesting a critical role for PtA L6b neurons in thalamo corticothalamic signaling.

### Somatosensory cortex

In the primary somatosensory cortex (S1), it has been clearly demonstrated that higher order thalamic nuclei such as the POm in the somatosensory system are innervated by L6b CT excitatory neurons (Bourassa et al., 1995; Killackey and Sherman, 2003). Using anterograde AAV tracing, these L6b CT neurons have been identified as *Drd1*-expressing PCs (Hoerder-Suabedissen et al., 2018). Only very few L6b CT neurons projected to the VPM (Casas-Torremocha et al., 2022). Furthermore, a large subset of L6b excitatory neurons in the S1 cortex in rat and mouse innervate different layers with their cortical area and project to adjacent cortical areas such as S2 and M1 cortex (Zhang and Deschênes, 1997; Zolnik et al., 2020); analogous observations have also been made for other sensory cortices (e.g., Prieto and Winer, 1999).

**FIGURE 6:**
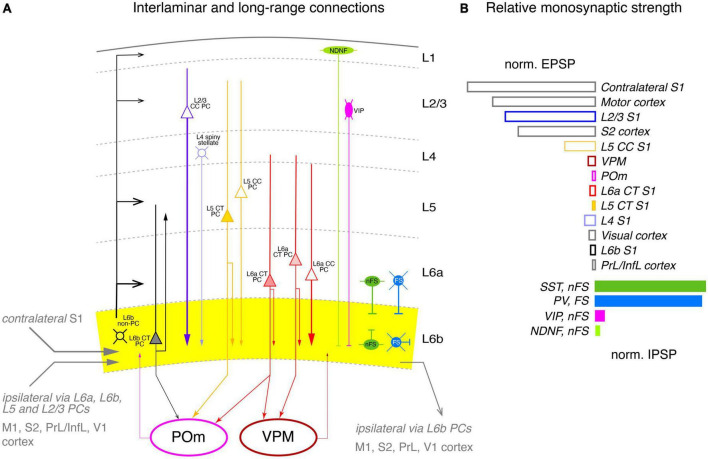
Intralaminar and long-range synaptic connectivity of layer 6b of the somatosensory cortex. **(A)** Simplified diagram of the S1 L6b synaptic input and output circuitry. L6b neurons are outlined in black, L2/3 PCs, in blue, L4 excitatory neurons in light blue, L5 PCs in yellow and L6a PCs in red. L1 NDNF interneurons are color-coded in light green, L2/3 VIP cell, magenta, L6 nFS (somatostatin + and neurogliaform) interneurons in green and L6 FS (parvalbumin+) interneurons in medium blue. Corticothalamic neurons in L5b and L6 are marked by a filled cell body. L6b provided synaptic input to all cortical layers but predominantly to layer 6a and 5b (as derived from the axonal projection pattern of L6b excitatory neurons); L6b CT neurons projects exclusively to the higher-order POm thalamic nucleus but receives little input from both POm and VPM, in contrast to layer 6a. Strong intrinsic excitatory input to layer 6b originates from L2/3, L5 CC and L6a CC neurons; strong inhibitory input comes mainly from nFS and FS interneurons within layer 6b and in adjacent layers 6a and 5 as the axonal domain of these interneurons suggest. Synaptic input and output to other cortical brain regions are given in gray. Arrow thickness signifies connection strength. Different arrow heads signify: ➤ corticothalamic connections from layers 5b and 6a; ▶ synaptic input to S1 layer 6b; >synaptic output from S1 layer 6b; inhibitory input is marked by bars. **(B)** Relative direct input strength (PSP amplitude × percentage of responding neurons) of the main pathways tested as revealed by ontogenetic photo stimulation of tagged neurons in different layers of the ipsilateral S1 cortex, other ipsilateral cortices and the VPM and POm nuclei. Modified from Zolnik et al. (2020) under a CC license.

The neuronal network providing synaptic input to layer 6b was only poorly understood until recently. Zolnik and coworkers identified the inter- and subcortical input to layer 6b in the primary somatosensory cortex using rabies monosynaptic retrograde tracing using *Drd1*-Cre and *Ctgf*-Cre neurons as starter cells (see Figure 6). Cell-type specific optogenetic stimulation was used in transgenic mouse lines in which specific (presynaptic) excitatory and inhibitory neuron subtypes are labeled (Zolnik et al., 2020). Postsynaptic L6b neuron types were identified according to marker genes (such as *Drd1*, *Ntsr1*, *Ctgf*), layer location and axonal projection pattern; however, it should be noted that these terminologies are not fully interchangeable.

As a first step, the authors examined morphological connectivity within the ipsilateral neocortex (Figure 6A). Intracortical excitatory presynaptic input to layer 6 was largely from neurons in layers 6a and 6b (together ∼45%), with substantial fractions from layers 2/3 and 5 (∼20% each); small fractions came from layers 4 (∼9%) and 1 (∼2%). However, layer 1 receives strong to very strong input from L6b CT (*Drd1+*) and CC (*Ctgf+*) excitatory neurons, respectively (Ledderose et al., 2023), but is also a target of TC afferents from the higher order thalamic POm nucleus (for a review see Schuman et al., 2021) to which the L6b (*Drd1+*) CT PCs project. This suggests that layer 6b is central to a L6b-POm-L1 corticothalamocortical loop which is independent of the “lemniscal” TC innervation via the VPM. Apparently, this connection is already established in early postnatal animals by *Cplx3*-expressing subplate/L6b neurons projecting to layer 1 (Viswanathan et al., 2017).

EPSPs in L6b excitatory neurons elicited by optogenetic stimulation of presynaptic L2/3 PCs were significantly stronger than those of L5, L6a, and L6b neurons; responses from L4 neurons were indirect, i.e., polysynaptic, possibly via recruitment of L2/3 PCs. Stimulation of both ispi- and contralateral L5 and L6 CT PCs resulted in only small EPSPs in layer 6b (Figure 6B).

The vast majority (95%) of the long-range intracortical afferents innervating layer 6b of S1 cortex came from the neocortex, with the largest proportion coming from the ipsilateral motor cortex, and here from layer 5; these inputs were particularly strong. Other cortical areas providing synaptic input to layer 6b were S2 cortex (9%), auditory cortex (7%), posterior parietal cortex (PPC, 2.5%), and visual and orbital cortex (<2% each). M1 and S2 inputs are already present in the subplate/layer 6b of the developing cortex and are likely to persist into adulthood (Tiong et al., 2019; Gellért et al., 2023). Low numbers of neurons presynaptic to layer 6b neurons were also found in the temporal, insular, cingulate, prefrontal, and perirhinal cortex (a region of the periallocortex that includes entorhinal and retrosplenial cortex). Consistent with the findings for PtA, L6b neurons were innervated to a considerable extent by axonal afferents from the contralateral hemisphere, mostly from somatomotor cortices. The largest fraction originated from the contralateral S1 cortex, mainly from deep layers 6a and 5 (58 and 29%, respectively) and layer 2/3 (13%). Another quarter of the contralateral presynaptic neurons were found in motor cortex and 8% in S2 cortex (Figure 6B).

Subcortical input (including TC connections) to S1 layer 6b was very sparse, accounting for only ∼1% of total presynaptic input. Most of the subcortical afferents originated in the thalamus, in particular from the second order nucleus POm, in line with previous findings in the rat showing that some axonal collaterals from this thalamic nucleus also ramify in layer 6b, albeit at a low density, but this was less than 1% of all presynaptic neurons, in line with the low density of POm and VPM axonal boutons in this layer (Meyer et al., 2010). Furthermore, the majority of L6b neurons showed little or no response to optogenetic stimulation of POm or VPM, with some of this input being indirect, i.e., not monosynaptic. In contrast, layer 6a showed a substantial and reliable response to VPM stimulation. This suggests that thalamic input to layer 6b of S1 cortex has little or no effect, in marked contrast to PtA, where activation of L6a neurons is enhanced by a synergistic effect of orexin and synaptic input from L6b excitatory neurons (see above; Hay et al., 2015).

### Entorhinal cortex

**FIGURE 7:**
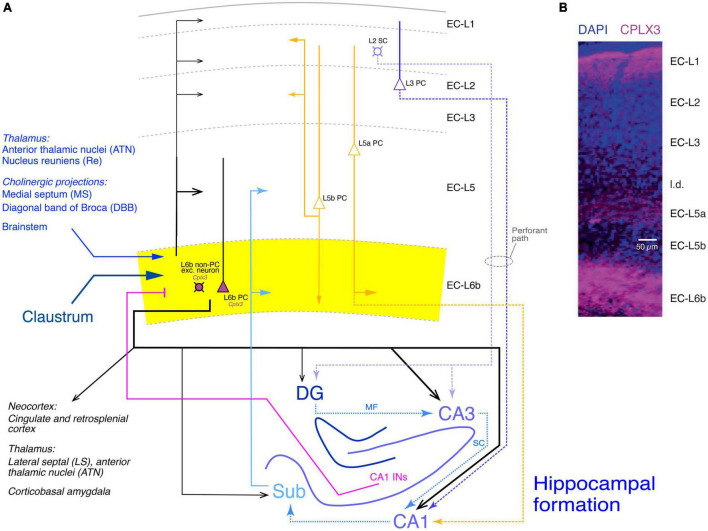
Synaptic connectivity of layer 6b of the entorhinal cortex (EC-L6b). **(A)** Simplified diagram of the EC-L6b synaptic input and output circuitry. EC-L6b neurons are outlined in black with purple cell bodies to indicate that they express *Cplx3*. EC-L6b excitatory neurons project through the EC and to the hippocampal formation, in particular to the CA3 region. To put the role of the EC-L6b to the hippocampus connections into context, the circuitry of the hippocampal formation is also shown in this figure. Different hippocampal regions, the excitatory axonal projections within the hippocampus, between EC-L2 and EC-L3 and the hippocampus (perforant pathway with EC-L2 to DG and CA3 and EC-L3 to CA1), and from the hippocampus back to EC-L6b are color-coded in different shades of blue. CA1 interneuron (CA1 INs) to EC-L6b projections are given in magenta. Other inputs to EC-L6b (from the thalamus, cholinergic regions, brainstem, and claustrum are shown in hues of dark blue. Furthermore, the recently identified L5a PC projections to CA1 (Tsoi et al., 2022) are also included and color-coded in yellow; EC-L5a PCs provide also the major intrinsic input to EC-L6b. Albeit closer to layer 6b, input from EC-L5b PCs to EC-L6b is sparse but strong in all other layers (Ohara et al., 2018). DG, dentate gyrus; Sub, subiculum; MF, mossy fibers; SC, Schaeffer collaterals. Arrow thickness signifies connection strength. Different arrow heads signify: ➤ synaptic connections not involving EC layer 6b; ▶ synaptic input to layer 6b; >synaptic output from EC layer 6b. **(B)** Layers of the entorhinal cortex (EC) revealed with DAPI and complexin 3 (CPLX3) immunostaining. the entire layer 6 is labeled by the subplate/L6b marker CPLX3 suggesting that entire EC-L6 corresponds to layer 6b in the neocortex. Modified after Ben-Simon et al. (2022) under a CC license.

The anatomical and functional synaptic connectivity of layer 6b has also been studied in the mouse entorhinal cortex (EC) which is not part of the neocortex proper but belongs also to the periallocortex which lies in the transition zone between the neocortex and hippocampus (Ben-Simon et al., 2022; see Figure 7A). This cortical area is a five-layered cortex and lacks a layer 4 proper. EC layer 6b neurons in the entorhinal cortex do not express marker genes specific for L6a neurons such as *Ntsr1* but rather subplate/L6b markers like *Cplx3* (Figure 7B), *Nxph4*, and *Ctgf* (Figure 7B). This suggests that EC layer 6 is more like neocortical layer 6b than layer 6a and will therefore be referred to as EC-L6b (Note: In Ben-Simon et al. (2022) the name for this layer is EC-6b; here, for consistency, the terminology EC-L6b has been adopted).

In this study, the expression of *Cplx3* has been used to label EC-L6b neurons (Figure 7B, *Cplx3*-expressing neurons are in violet). Retrograde rabies virus tracing revealed that EC-L6b neurons predominantly innervate hippocampal CA3 PCs, in addition to the canonical (perforant) pathway from EC-L2 to CA3 and the recently identified L5a PC - CA3 connection (Tsoi et al., 2022). This adds another layer of complexity to the hippocampal neuronal network.

EPSCs resulting from the perforant path or mossy fiber projections (i.e., EC-L2-CA3 and DG-CA3 connections, respectively) are characterized by a fast time course and short-term synaptic facilitation. In marked contrast, EPSCs elicited by optogenetic EC-L6b stimulation have a significantly slower time course and showed almost complete short-term depression indicating that the synaptic release probability at this connection is high. This establishes the EC-L6b connections as a source of substantial excitatory input to CA3 PCs. The EC-L6b-to-CA3 connection may play a role in spatial coding, learning and memory as the decreased performance in spatial orientation and learning following optogenetic inactivation of excitatory EC-L6b neurons suggests (Ben-Simon et al., 2022).

Apart from the strong EC-L6b-CA3 connection, EC-L6b excitatory neurons provide strong synaptic input to CA1 PCs; in addition, dentate gyrus granule cells (DGCs) and hippocampal interneurons are also sparsely innervated by EC-L6b. This suggests that EC-L6b neurons are presynaptic to all hippocampal regions with a preference for CA3 and to a lesser extent CA1. Furthermore, EC-L6b axonal projections were found to target the anterior thalamic nuclei (ATN) and the lateral septal nucleus (LS).

Prominent excitatory synaptic input to EC-L6b was found to originate from the claustrum (Cla) which may share a common developmental origin with the subplate/L6b (Bruguier et al., 2020). On the other hand, only sparse input was found from the cingulate cortex (Cg), the retrosplenial cortex (RSC) and the corticobasal amygdala (CoA). Additional axonal projections to EC-L6b neurons come from the thalamic nucleus reuniens (Re) and the anterior thalamic nuclei (ATN) as well as the medial septum (MS)/diagonal band of Broca (DBB), both of which provide glutamatergic, GABAergic and cholinergic input to the hippocampus.

From the hippocampus, EC-L6b neurons receive excitatory input from the CA1 region and the subiculum, and inhibitory input from a population of CA1 stratum oriens and radiatum interneurons. Finally, major intrinsic excitatory EC synaptic connections originate predominantly from layers 5a and 6b itself, with other layers such as layer 5b providing low density input (see also Ohara et al., 2018).

## Neuromodulation of layer 6b neurons

Layer 6b is under the strong influence of neuromodulatory transmitters, many of which are neuropeptides. These neuromodulators are released from various subcortical brain regions and profoundly and often selectively alter L6b synaptic signaling. L6b neurons express many neuropeptide receptors, but are also strongly affected by acetylcholine and monoamines such as norepinephrine and dopamine. Here I will mainly discuss those neuromodulatory systems in layer 6b that have been investigated in some detail.

### Orexin/hypocretin

The most prominent and best studied example of L6b-specific neuromodulation is that of the neuropeptide orexin/hypocretin. Orexinergic neurons are a subset of neurons located in the lateral hypothalamus that give rise to diffuse projection to many subcortical regions including the basal forebrain, the locus coeruleus and the entire cerebral cortex. Orexin is involved in the regulation of appetite, sustained arousal and wakefulness (De Lecea et al., 1998; Jones, 2008). Direct evidence that orexinergic neurons promote the transition from sleep to wakefulness was provided by selective, optogenetic photostimulation of these cells in the lateral hypothalamus (Adamantidis et al., 2007), probably by inhibiting sleep (De Luca et al., 2022). Orexin has been implicated in many neuropsychiatric disorders such as narcolepsy, epilepsy, drug addiction, eating disorders, as well as schizophrenia, Parkinson’s disease and neurodegenerative disorders (Ten-Blanco et al., 2023).

There are two types of orexin, orexin-A (hypocretin-1) and orexin-B (hypocretin-2), which bind predominantly to the two types of orexin receptors, the G-protein coupled type 1 (OX_1_R) and type 2 (OX_2_R) receptors, respectively. Early studies revealed that OX_2_Rs were highly expressed in neocortical layer 6b, but were also found in other cortical layers (Marcus et al., 2001; Cluderay et al., 2002) whereas OX_1_Rs were not present in this layer (Hervieu et al., 2001).

Bath-applied orexin-B causes a strong depolarizing response in L6b excitatory neurons–both PC and non-PC neurons–mediated by OX_2_Rs. OX_2_Rs are exclusively expressed in L6b excitatory neurons of sensory, motor, prefrontal or frontal cortices (Bayer et al., 2004; Hay et al., 2015; Wenger Combremont et al., 2016b) but not in L6b interneurons or neurons in other neocortical layers. In L6b but also in L5 FS interneurons, orexin caused an increase in the spontaneous EPSP/EPSC frequency; this effect is indirect and mediated through activation of presynaptic boutons from L6b excitatory neurons (Aracri et al., 2015; Wenger Combremont et al., 2016a). It has been suggested that the main target neurons of orexin modulation in layer 6b are multipolar spiny neurons located close to the white matter, suggesting a cell specificity of the orexin effect (Wenger Combremont et al., 2016b). However, because an orexin-B response has been demonstrated in PtA L6b CT PCs, this seems unlikely. In these L6b neurons, orexin plays a role in a feed-forward excitatory pathway between L6b and L6a CT PC (see above and Figure 5; Hay et al., 2015). This orexin-B response is potentiated by activation of nicotinic acetylcholine receptors (nAChRs) suggesting a synergistic interaction between orexin and ACh and possibly other neuromodulators.

Finally while only L6b excitatory neurons respond to orexin-B, a depolarizing response to orexin-A has been observed in L2/3 PCs in rat PFC (Yan et al., 2012) and L5 PCs in mouse prelimbic cortex (Li et al., 2010).

### Cholecystokinin

Cholecystokinin (CCK) was initially thought to be a gut peptide, with one of its primary roles being the regulation of digestion and food metabolism; it is found at high levels in the gastrointestinal tract. However, it was later discovered that CCK is even more abundant in the mammalian brain, particularly in the neocortex (Rehfeld, 2017, 2021). CCK acts through two G protein-coupled receptor types, the CCK_*A*_ and CCK_*B*_ receptors (CCK_1_ and CCK_2_, respectively).

The *CCK/Cck* gene is highly expressed in both hippocampal and neocortical excitatory neurons and GABAergic interneurons and here mainly in one specific interneuron subtype, the non-FS, CCK-positive large basket cell (Kawaguchi and Kondo, 2002; Whissell et al., 2015). *Cck*-expressing excitatory neurons have also been identified in layer 6b; these neurons resemble the multipolar cell type and have been found close to the white matter (Gallopin et al., 2006). Furthermore, *Cck*-positive axons have also been identified in corticothalamic projections from deep cortical layers (Senatorov et al., 1995; for reviews see Beinfeld, 2001; Lee and Soltesz, 2011).

CCK_*A*_ and CCK_*B*_ receptors have been found in deep neocortical layers PCs, in the amgydala, hippocampus, and thalamus (Schiffmann and Vanderhaeghen, 1991; Honda et al., 1993; Mercer et al., 2000; Mercer and Beart, 2004; Andjelic et al., 2009). Application of CCK resulted in long-lasting depolarizations in rat somatosensory cortical L5b, L6a, and L6b excitatory neurons, i.e., the layers from which CT afferents originate. This effect was weak in layers 5b and 6a, but robust in layer 6b, where sustained AP firing was observed in ∼50% of L6b excitatory putative CT neurons. The CCK response was mediated by activation of CCK_*B*_ receptors resulting in decreased voltage-independent K^+^ leak current (Chung et al., 2009). However, the role of CCK neuronal signaling pathways in the neocortex, and in particular layer 6b, remains to be elucidated.

### Neurotensin

Neurotensin is involved in the regulation of sleep patterns, locomotion, body temperature, and food and fluid consumption (Leinninger et al., 2011; Gereau et al., 2023). Neurotensin is released from several subcortical areas including the lateral hypothalamus where it is involved in the control of orexinergic neurons (Brown et al., 2018; Brown et al., 2019) and midbrain dopaminergic neurons (Piccart et al., 2015). Neurotensin acts through two G protein-coupled receptors, NTSR1 and NTSR2. The *Ntsr1* gene has been localized to L6 CT neurons that project either to the VPM alone or to both the VPM and POm. Therefore, *Ntsr1* has been used as a genetic marker for these neurons in transgenic mouse lines.

In mouse somatosensory cortex, slow-bursting multipolar L6b neurons similar to those that show a depolarizing responses to orexin and CCK, exogenously applied neurotensin produces a strong depolarizing response (Wenger Combremont et al., 2016b) that often results in prolonged AP firing, as do the monoaminergic transmitters dopamine, histamine and noradrenaline which are also involved in arousal. In L6b FS interneurons, neurotensin produced a strong increase in the frequency of spontaneous EPSPs, an effect that was probably induced through activation of presynaptic L6b excitatory neurons. Thus, neurotensin enhances the activity of L6b excitatory and inhibitory neurons, thereby contributing to the transition to wakefulness and attention.

### Acetylcholine

Acetylcholine (ACh) is a classical neurotransmitter that is released from cholinergic neurons in the nucleus basalts of Meynert in the basal forebrain, which innervates every cortical area. ACh release is involved in wakefulness, arousal, REM sleep and attention (Picciotto et al., 2012). Dense cholinergic axonal projections can be found in all layers of the neocortex, with particularly high axonal bouton densities in layers 6, 5, and 1 (Eckenstein et al., 1988; Henny and Jones, 2008; Kalmbach et al., 2012). In addition, ACh is also co-released with GABA from a subset of vasoactive intestinal peptide (VIP)-expressing interneurons, which are predominantly located in cortical layer 2/3 (Obermayer et al., 2019).

ACh acts through both muscarinic, G protein-coupled receptors (mAChRs) and nicotinic receptor channels (nAChRs). Information regarding mAChRs in layer 6b is scarce so that I will concentrate on nicotinic effects on L6b neurons. A cholinergic modulation of L6b neurons has already been suggested in early studies showing that the level of acetylcholinesterase was high in layer 6b (Kristt, 1979) and that in both rat and mouse the situ hybridization signals for the nAChR α_4_, α_5_, and β_2_ subunit genes (*Chrna4, Chrna5*, and *Chrnb2*, respectively) were strong (Wada et al., 1989; Wada et al., 1990; Belgard et al., 2011). On the cellular level, *Chrna4, Chrna5*, and *Chrnb2* expression has been identified primarily in rat L6b excitatory neurons (Bravo and Karten, 1992; Ostermann et al., 1995; Hay et al., 2015). The majority of rodent L6b neurons is excited by acetylcholine and nicotinic agonists through the activation of heteromeric nAChRs containing α_4_, α_5_, and β_2_ subunits, i.e., α_4_β_2_α_5_ nAChRs (Kassam et al., 2008; Proulx et al., 2014; Hay et al., 2015; Venkatesan and Lambe, 2020). Importantly, the α_5_ subunit confers a high acetylcholine sensitivity (EC_50_ ∼1 μM) to α_4_β_2_ heteromeric receptors (Ramirez-Latorre et al., 1996; Kuryatov et al., 2008).

The ACh response in L6b excitatory neurons is strong and can lead to sustained AP firing but only when orexin-B is present (see above). This suggests an intracortical synergistic link between the orexin system and cholinergic modulation; it has been proposed that this mechanism serves to promote the transition to wakefulness and vigilance (Hay et al., 2015; Villano et al., 2017). More recently, recording from a group of subplate neurons have revealed a population of so-called “ACh super-responders” (Venkatesan et al., 2023) with unusually strong ACh responses that almost invariably elicit AP firing. Whether these subplate neurons are precursors of an L6b excitatory neuron subpopulation in the adult remains, however, to be determined.

### Dopamine

In the neocortex, dopamine is released by axonal afferents from the substantia nigra (pars compacta, SNc)-ventral tegmental area (VTA) complex (Swanson, 1982; Oades and Halliday, 1987; Haber, 2014). Dopaminergic innervation is most dense in layer 1 and deep cortical layers. However, it is significantly stronger in primates than in rodents (Albanese et al., 1986; Descarries et al., 1987; Berger et al., 1991; Bentivoglio and Morelli, 2005). Dopaminergic neurons in the SNc-VTA complex that project to the neocortex have either mesocortical or mesocorticolimbic axons, of which only the latter also innervate limbic structures such as the amygdala. Mesocortical axons appear to branch extensively in the neocortex, whereas mesocorticolimbic axons are significantly less dense but still innervate layer 6b (Aransay et al., 2015).

Dopamine acts through two types of receptor, the D1-like receptors (comprising dopamine receptor 1, DRD1 and DRD5) and the D2-like receptors (DRD2-4) which are coupled to excitatory and inhibitory G-proteins (G_*s*_ and G_*i/o*_), respectively. D1-like receptors are prominently expressed in deep cortical layers including layer 6b (Berger et al., 1991).

As mentioned above, the *Drd1* gene is highly expressed in L6b CT PCs (Hoerder-Suabedissen et al., 2018) but also in other L6b excitatory neurons. In L6b multipolar excitatory neurons, application of dopamine (10 μM) results in a depolarization that often develops into prolonged AP spiking (Wenger Combremont et al., 2016b), suggesting that these neurons are highly sensitive to modulation not only by ACh but also by dopamine. Whether POm-projecting *Drd1* L6b PCs and/or other L6b excitatory and inhibitory neurons are susceptible to dopaminergic modulation is so far not known.

### Other neuromodulators

L6b neurons are also susceptible to modulation by several other neurotransmitters such as the monoamines noradrenaline and histamine which have been shown to enhance the activity of L6b excitatory neurons and FS interneurons. In addition, it would be interesting to see whether other neuropeptides involved in the sleep/wake cycle or food intake and digestion such as neuropeptide Y (NPY) act as neuromodulators in layer 6b, the gene of which is highly expressed in both adaptive spiking and FS L6b interneuron types (Perrenoud et al., 2013); however, it is not clear whether NPY is released from L6b interneurons and how this would affect L6b excitatory neurons because the effects of NPY on neocortical synaptic transmission appear to be complex (Bacci et al., 2002).

### Interactions of neuromodulatory systems

**FIGURE 8:**
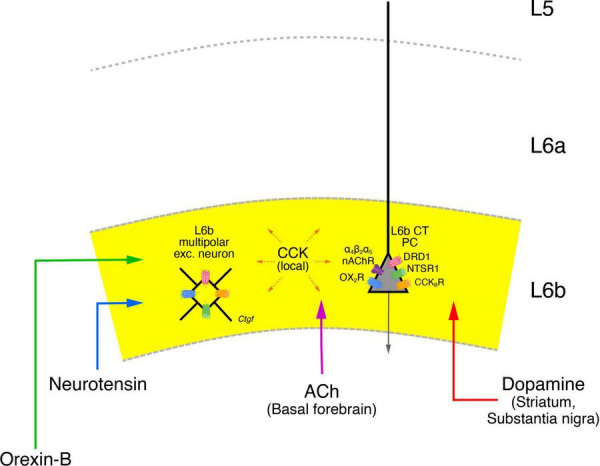
Neuromodulatory influence on layer 6b neurons. Layer 6b is under tight control of neuromodulators such as the neuropeptides Orexin-B (green), Neurotensin (blue), Cholecystokinin (CCK, orange) as well as acetylcholine (ACh, violet) and dopamine (red) all of which produce direct depolarizing responses in L6b excitatory neurons. The brain regions from which a particular neuromodulator is released are given in brackets under the name of the neuromodulator. The brain regions from which orexinergic and neurotensinergic afferents entering layer 6b originate are not clearly known and have therefore been omitted here. CCK is released from neurons in the neocortex both in layer 6b but also from other cortical layers. The names of the neuromodulator receptors are given next to the receptor drawing; apart from the nAChRs all receptor types are G-proteins (see text for details). The release of orexin in the lateral hypothalamus is under the control of neurotensin which promotes their activity. Orexinergic neurons control the release of ACh and dopamine in the basal forebrain and the VTA-SNc complex, thereby exerting also an indirect control over cortical activity, in addition to the more direct modulatory role in layer 6b. In addition, cholinergic neurons in the basal forebrain are also innervated by VTA-SNc dopaminergic neurons; these neurons receive also cholinergic input put from brainstem nuclei and not the nasal forebrain. These and other details in the network of the neuromodulatory systems shown here have omitted for clarity.

Orexin appears to coordinate the release of several of the L6b targeting neurotransmitters/neuropeptides. In particular, the dopaminergic VTA-SNc complex is densely innervated by orexinergic neurons from the lateral hypothalamus (Bentivoglio and Morelli, 2005; Del Cid-Pellitero and Garzon, 2014; Thomas et al., 2022), suggesting that in addition to the direct orexinergic modulation of L6b neurons, this neuromodulator also acts indirectly by controlling cortical dopamine release. Furthermore, cholinergic neurons in the basal forebrain are also densely innervated by orexinergic neurons in the lateral hypothalamus; they have been shown to promote ACh release in the neocortex via OX_2_R activation (Eggermann et al., 2001; Arrigoni et al., 2010; Gotter et al., 2016; Villano et al., 2017; see Figure 8). Finally, dopaminergic neurons in the VTA-SNc also project to the basal forebrain where they target cholinergic neurons (Fallon and Moore, 1978; Fallon and Loughlin, 1982; Yetnikoff et al., 2014). The activity of these dopaminergic neurons is also regulated by ACh released from the cholinergic hindbrain nuclei, the pedunculopontine tegmental nucleus and the laterodorsal tegmentum (De Kloet et al., 2015). A more detailed description of this modulatory network is, however, beyond the scope of this review.

Thus, the orexin-neurotensin system is involved in the activation of cholinergic neurons in the basal forebrain and dopaminergic neurons in the VTA-SNc complex which show also direct interactions with one another. All of these systems appear to converge in layer 6b, making this layer a tightly controlled system. It has been argued that like L5b CT PCs, L6b CT neurons are also drivers of the POm (Ansorge et al., 2020). However, given that L6b activity itself is under the control of the neuromodulatory systems described above and probably several more, it seems more likely that L6b CT neurons either act as modulators of POm or switch between a “modulator’ and a “driver” state depending on the behavioral context.

## Conclusion

Layer 6b consists of a dense and long-range neural network that is highly specific for a given brain region. It integrates input from multiple cortical and/or subcortical regions and projects widely to adjacent areas in the neocortex and, in the case of the entorhinal cortex, to the hippocampus. Layer 6b is strongly modulated by several neuromodulatory transmitters and neuropeptides, all of which are released during different behaviors such as arousal and food intake. Further work is needed to dissect the input and output neuronal circuitry of layer 6b neurons in specific cortical areas, their similarities and differences, and to determine which neuromodulatory systems control their activity both *in vitro* and *in vivo* in order to understand the functional role of layer 6b in the neocortex of rodents, humans and other animals.

## Author contributions

DF: Conceptualization, Funding acquisition, Writing—original draft, Writing—review & editing.

## Footnotes

1
http://celltypes.brain-map.org/data
